# The Case for Making Health Care Advocacy a Discipline of Medicine; The Paradigm of a Vascular Patient

**DOI:** 10.3389/fsurg.2015.00033

**Published:** 2015-08-14

**Authors:** Elias J. Arbid, Ibrahim G. Eid

**Affiliations:** ^1^Section of Vascular and Endovascular Surgery, Lebanese American University, Beirut, Lebanon; ^2^Division of Vascular Surgery, Primacare Center for Vascular Diseases, Fall River, MA, USA

**Keywords:** health advocacy, physicians health advocates, patient centered care, vascular surgery, turf battles

The time has come for the medical profession to embrace health advocacy as a new discipline of medicine. When facing issues like cancer or major surgery, patients often seek second opinions, and as they do, the second recommendation is likely to differ from the original one in around 30% of the cases (1). In numerous studies about Decision Aid Support, patients given a brochure to read about their choices tend to opt out of surgeries in at least 20% of the times (1). These studies were conducted in centers of excellence, where abuse and over utilization are assumed to be minimal. When patients are given credible education, patients often steer their care from the direction chosen by their treating physician (1).

It is naïve to think that financial gains are the only factors that influence a physician’s recommendation. “High volume” physicians gain respect in the medical community and get direct and indirect power as they assume leadership roles in hospitals and medical groups. In the case of surgical specialties, the so-called “easy” cases that usually lead to good outcomes are good statistics for a particular surgeon, as they provide the denominator over which complications are counted. Indeed, these easy and safe cases do not give an uninformed patient a complete picture, and surely add to the inflated national health care bill.

One would assume that the health care reform with the pay for performance schemes and the return of managed care could protect consumers and patients from non-necessary care but this does not undo the incentives mentioned above, and the opposite may be happening in some cases. Hospital systems are buying physician practices at unprecedented rates and physicians are now incentivized to “feed” patients to the expensive hospital programs and “cost centers.” In pure capitation models, patients may suffer from under-utilization as the system is now incentivized to reduce expenditure (2). In addition, health care systems, in an effort to cut cost, are often opting to become limited networks and patients may never be informed about care options that may suit them better and that are outside of their network (3, 4). The need for health advocacy has never been more acute.

It is disappointing that the medical profession has failed to recognize that the private sector is more than ever aware of these problems and has already taken consumer protection measures aimed at health care as a sold commodity. Health advocacy is a billion dollar industry serving employers, consumers, and insurance companies alike, and has been populated by nurses, social workers, and now computer software, but rarely physicians. Health advocacy will, however, never prove its full value until it is delivered by the source of health care knowledge, the physician. Many studies support what we all know: when patients are facing major health decisions, the most powerful source of decision making remains the physician, followed by friends and family, and the “human” source of information beats the internet and what is in print (1). When patients seek information from various websites available on the internet, they are exposed to a plethora of information that is often simplified for the reader to understand and, in many instances, does not address the complex issues related to the care of a particular vascular patient. The information is disease specific but not patient specific. Indeed, patients may not be able to evaluate the quality of the printed information available on different web sites, and how such information, treatment modality, or stated outcome applies to their particular case. Patients often need guidance and counseling to synthesize all that information, and providing them with the right knowledge in the right context for them to make the right decision is what health advocacy is all about. It is therefore essential that physicians understand how to engage their patients in the decision-making process, but more importantly, physicians need to do so in a way that the patient, the consumer, considers to be meaningful and helpful.

In 2007, Fraenkel and McGraw examined the elements necessary for patients to become fully engaged in their healthcare processes (5). In that study, patients noted five qualities as essential qualities to foster by health care advocates:

1. Patient knowledge: patients emphasized that being adequately informed and able to fully process the amount and complexity of information involved was essential.
2. Explicit encouragement of patient participation by physicians: patients desired that physicians facilitate the shared participation by inviting questions to be asked.
3. Appreciation of the patient’s responsibility/rights to play an active role in decision making: physicians must respect the desires and rights of the patient to make decisions.
4. Awareness of choice: patients must understand that there is uncertainty in medicine and choices are available.
5. Time: enough time must be available between physician and patient for proper discussion.

Case in point, vascular health and wellness is an area of medicine that involves care by many specialists who are often in a competitive relationship. Take, for example, the case of a diabetic patient with heart disease and peripheral arterial disease who has developed critical ischemia and tissue loss in their foot. They may search for treatment on a variety of websites starting with general knowledge websites, to specialty websites, to industry websites. These sites will discuss the condition in detail, but very few, if any, will present the patient with a comprehensive treatment approach that takes into account their diabetes, heart disease, podiatry needs, surgical and interventional needs, and more importantly discuss the various health outcomes related to any potential treatment or intervention. It is imperative that the patient not only understands the effects of any potential intervention on the foot or leg but also how such intervention may impact all the other medical conditions he or she suffers from. Such understanding has to come before the patients get in the system, as it becomes almost impossible to remain objective and make the right choice for themselves when they become caught in the gearing of a health care system, with preset referrals. In real life, patients may not have access to all the expertise available within an institution, and having a toxic relationship among specialists undermines the concept of a second opinion within the same community, since it is often provided by a competitor rather than a collaborator. None less than a medical professional, trained in the discipline of health advocacy, will empower the patient to question his treating physician and mold his care to his preferences and values.

Consequently, it makes perfect sense that physicians themselves should be the primary advocate for their patients by providing the knowledge to make decisions, both at the medical level and, equally importantly, at a consumer level. As patients come to realize that not all physicians are trained the same, they should be empowered to ask questions not only about medical choices, but also about their physicians, board certification and accreditation status of the specialist, the hospital’s volume, outcomes, and last but not least, costs. In the case of the patient hereto mentioned, the opinions regarding what the “best treatment would be” and the outcomes of such treatment may vary whether the patient seeks the advice of a cardiologist, podiatrist, vascular surgeon, or even their primary care physician. An interventional radiologist or cardiologist will often highlight the benefits of an endovascular approach which they are trained to perform and strongly recommend it over an open vascular approach when, indeed, the latter may be the preferred treatment for that particular patient, and vice versa. Yet, these differences are not mere differences in opinions among specialists, but are rooted in deeper philosophical differences among the various professional medical societies. In real life, there are clear differences among how the specialty societies view a particular condition, a particular treatment, or in what they might consider an acceptable outcome. The level of evidence sought by various societies for a particular treatment varies, the way studies are conducted varies, and the scrutiny of outcomes by various societies of their members varies. An inter-societal collaboration has emerged to try and contain such differences and to come up with unified guidelines. Unfortunately, these guidelines are not widely disseminated, and they remain elusive in a number of countries around the world where they may be unknown or simply ignored.

These turf battles for the care of vascular patients are present in every institution, medical center, and hospital, albeit in varying forms and to different degrees (Figure 1).

**Figure 1:**
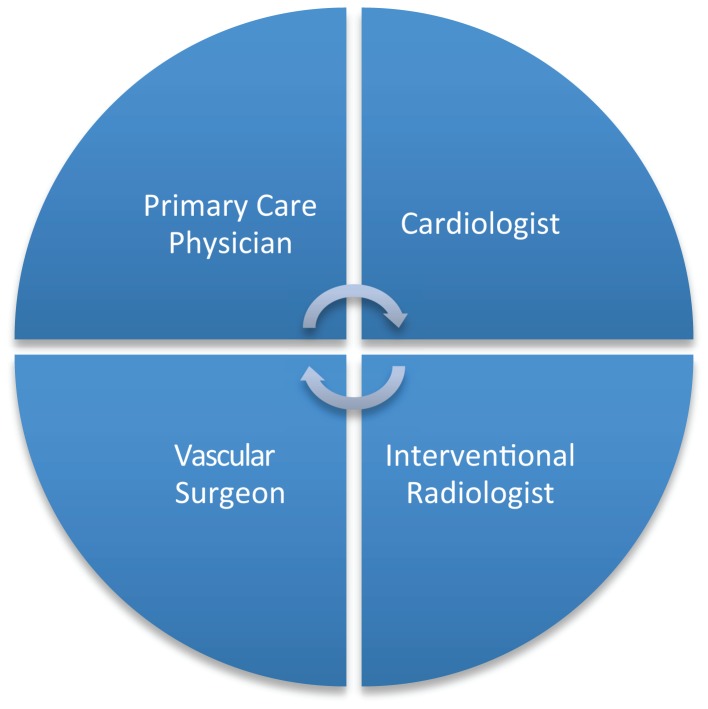
**Turf battle among various specialists involved in the care of a vascular patient may deny the patient the full expertise available at an institution**.

Sadly, these turf battles are by in large driven by financial incentives. This is why they are often won not by merit but by the financial impact a particular physician or group has in the medical community. Fortunately, many hospitals have been able to bridge the gap between physicians, and have used a variety of tools like financial incentives, laying down rules, and forming joint committees and councils to mitigate specialist rivalries. As a medical community or institution sets the grounds for collaboration rather than competition, physicians can now be health advocates, and a number of these fore-mentioned problems will be solved to the best of patients. The time is not far when, patients, insurance providers, and government regulations will demand the collaboration of the various specialists, and we better be ready and set to respond. Information technologies have certainly shortened that time, necessitating that we provide patients, and the public at large with the right education as to what is that we do, how we do it, and last but not least, how well we do it.

It remains the issue of how to engage in effective healthcare advocacy. Many organizations have developed health advocacy committees. Others may not have an advocacy committee up and running in their organization but would like to form one. The following principles may be helpful:

1. Focus on patients: effective advocacy focuses on preserving and broadening access to services for patients. The goal of these efforts is to maintain and expand access to medically necessary healthcare. This is important, because advocacy efforts based on preserving vascular surgeons, cardiologists, and radiologists’ income, while important, should not be the primary concern for health advocates.
2. Build coalitions with other health care providers. Undoubtedly, there are willing partners in each institution: reach out to them and start the discussion.
3. Form a cardiac and vascular advocacy committee in your hospital, if one does not yet exist.
4. Replicate success. Watch what other hospitals or organizations are doing, and replicate success. In one such model, having a cardiac and vascular coordinator who triages patients and directs them to the appropriate specialist, and follows them through their treatment fulfills the role of the health advocate very nicely while maintaining independence of the various specialists (6).
5. Do not be discouraged if this seems overwhelming; once you get started, information, experience, and support from others will fuel your efforts.

Remember that it is all about the patient. Active and effective communication between the patient, their physician, and the organization where they are receiving care remains the cornerstone of any health care advocacy model.

In short, the primary goal of health care advocacy is to support the patient’s ability to understand and act on health information. Until health advocacy shapes up as a discipline of medicine, where a physician can dedicate time to learn the skills necessary to provide information support and gets reimbursed separately for that important function, matching the right treatment to the right patient in the right setting of care will remain as accurate as a roll of a dice.

## Conflict of Interest Statement

The authors declare that the research was conducted in the absence of any commercial or financial relationships that could be construed as a potential conflict of interest.
